# Τemporal Variation in Pesticide Residues in Citrus Fruits from Chios, Greece, before and after the Development of an Integrated Pest Management Strategy (IPMS): A Five-Year Study (LIFE13 ENV GR/000414)

**DOI:** 10.3390/toxics9120323

**Published:** 2021-11-29

**Authors:** Eleftheria Bempelou, Christos Anagnostopoulos, Maroula Kiousi, Panagiota Malatou, Konstantinos Liapis, Nikos Kouloussis, Vassilis Mavraganis, Nikolaos T. Papadopoulos

**Affiliations:** 1 Pesticide Residues Laboratory, Benaki Phytopathological Institute, St. Delta 8, Kifissia, 14561 Athens, Greece; c.anagnostopoulos@bpi.gr (C.A.); m.kiousi@bpi.gr (M.K.); p.malatou@bpi.gr (P.M.); k.liapis@bpi.gr (K.L.); 2 Laboratory of Applied Zoology and Parasitology, School of Agriculture, Aristotle University of Thessaloniki, 54124 Thessaloniki, Greece; nikoul@agro.auth.gr; 3 Institute of Soil and Water Resources, HAO-DEMETER, S. Venizelou 1, Lykovrissi, 14123 Athens, Greece; mavrag1a@otenet.gr; 4 Laboratory of Entomology and Agricultural Zoology, Department of Agriculture Crop Production and Rural Environment, University of Thessaly, Phytokou St., 38436 Volos, Greece; nikopap@uth.gr

**Keywords:** pesticide residues, plant growth regulators, citrus fruits, Integrated Pest Management Strategy (IPMS), food safety

## Abstract

The temporal variation in pesticide residues in Kampos, of Chios Island, in Greece, was determined between June 2014 and October 2019. Monitoring of residues took place before and after the development of an Integrated Pest Management Strategy (IPMS) for the sustainable control of the Mediterranean fruit fly (medfly) based on mass trapping with the non-toxic and environmentally friendly attractant Biodelear. A total of 1252 samples of citrus fruits, collected from 12 experimental citrus orchards, were analyzed for the presence of 353 active substances and metabolites of pesticides. A modified QuEChERS method and sensitive chromatographic techniques were used. During preparatory monitoring for the project, the most frequently detected pesticides were the insecticides chlorpyrifos, deltamethrin and spirotetramat; the fungicides propamocarb, dimethomorph and mepanipyrim; and the synergist piperonyl butoxide. The implementation of the IPMS to address medfly resulted in a dramatic reduction in the pesticides detected in citrus fruits during confirmatory monitoring, with no detectable residues—which may cause serious problems to human health—in any of the samples analyzed at the end of the project, thus enhancing consumer safety.

## 1. Introduction

Nowadays, pesticides are used intensively in the primary agricultural sector to control infestations of insects, microorganisms, fungi and weeds [1,2]. Pesticides may enter into the human organism through inhalation, dermal uptake or through food consumption [3]. Undoubtedly, farmers and, especially, operators run the highest risk of exposure to pesticides; however, consumers are exposed to pesticides through the diet and water [4,5], whereby pesticides enter into the food chain, potentially causing serious problems for human health, even in low quantities. The intensive application of pesticides, either as products for plant protection or as biocides, affects the quality of the environment in agroecosystems with respect to food safety and environmental parameters.

Citrus is one of the most economically important and popular fruit crops worldwide. Historically, it is believed that *Citrus medica* L. (citron) was the first citrus tree to be recorded in Europe and the Middle East, and was imported from India into Greece, Turkey and North Africa by Alexander the Great in the late 4th century BC [6]. During 2019, the world production of citrus fruits was estimated to be approximately 180 million tons, 24 million tons of which was in the Mediterranean Basin, with approximately 1 million tons being in Greece [7]. Approximately 15–20% of the global citrus production is situated in the Mediterranean Basin, as well as approximately 60% of the global fresh citrus trade [8].

There is a wide range of species of fungi and insects that infest citrus crops, among which we find the Mediterranean fruit fly (medfly) (*Ceratitis capitata*, Diptera: Tephritidae). It is considered to be the most common pest in citrus orchards in the Mediterranean Basin, causing irreversible damage in citrus fruits, leading to important losses in crop yields. To date, the main approaches recommended to control medfly include bait spray applications in the foliage, mainly used to control the adult population, and cover sprays in the entire tree canopy, also to control adult medfly, as well as to provide limited efficacy in the control of eggs and larvae [9]. Consequently, significant residues of pesticides may often be left on citrus fruits and, even when humans consume nutritious food, potential health hazards may be present.

Monitoring programs have been organized and implemented all over the world to certify conformity with the Maximum Residue Limits (MRLs) of pesticides and to estimate consumer safety from residues. In Greece, the evaluation and monitoring of pesticide residues in products of plant origin is administered by the Ministry of Rural Development and Food, within the framework of the official control activities on pesticide residues of the European Union (EU). Quality standards for citrus fruits in the form of MRLs with respect to the presence of a single pesticide are set by Regulation 396/2005 [10]. High variation is observed in the levels of the corresponding MRLs, which range from 0.01 mg/kg for diflufenican [11] to 5 mg/kg for imazalil [12]. According to the official database of the Ministry of Rural Development and Food [13], a total of 419 plant protection products (ppps) (containing 82 different active substances) have been authorized in Greece in oranges and mandarins. Of these, a total of 169 ppps (containing 35 different active substances) are insecticides.

A number of studies have investigated the quality of citrus fruits with respect to pesticide residues. A plethora of analytical methods have been developed for the identification of pesticide residues in citrus fruits, using techniques such as gas chromatography [14,15,16,17,18,19,20], liquid chromatography [19,21,22,23,24,25,26,27,28,29], immunoassay [30] or by nanoplasmonic sensor array [31].

The present research took place during the implementation of the project LIFE-BIODELEAR (LIFE13/ENVGR/000414), entitled “*Addressing Med fly with an innovative friendly attractant through an Integrated Pest Management Strategy*”, and, for the first time, presents a five-year study on the temporal variation and distribution of residues of ppps, before and after the development and implementation of an Integrated Pest Management Strategy (IPMS) for the sustainable control of the medfly, mainly through mass trapping using the non-toxic and environmentally friendly attractant Biodelear. The efficacy of mass trapping with the attractant Biodelear was evaluated by comparing the *Ceratitis capitata* population level, the fruit infestation rate and the ground biodiversity of arthropods. Apart from the development of a sustainable IPMS at local/regional scale, the project also focused on the exploitation and valorization of ecosystem indicators before and after the implementation of the strategy. The project took place in the Kampos region of Chios Island in Greece. Kampos is a unique territory, well-known for its citrus orchard architecture. It is centrally located on the island of Chios and has been characterized by the Ministry of Culture as a “historic site”. The region was developed as an urban area around the castle of Chios by the Genoese during the 14th century, a date that also heralded the introduction of citrus trees to the island [32,33]. This area was selected for the field experiments as it is highly regarded as a unique and protected environment.

Citrus fruit samples from 12 experimental orchards were analyzed for ppps and plant growth regulators (pgrs), including metabolites and degradation products, using two multiresidue analytical methods based on QuEChERS, followed by liquid and gas chromatography analysis. In this study, the determination of residues acted as a tool for evaluating the effectiveness of this innovative attractant in the control of medfly, while also depicting the environmental fingerprint of residues from pesticides in the citrus orchards of Kampos, Chios.

## 2. Materials and Methods

### 2.1. Studied Area

As already reported, all the samples analyzed in the present study originated from citrus orchards in the area of Κampos, which is on the island of Chios, Greece.

**Figure 1:**
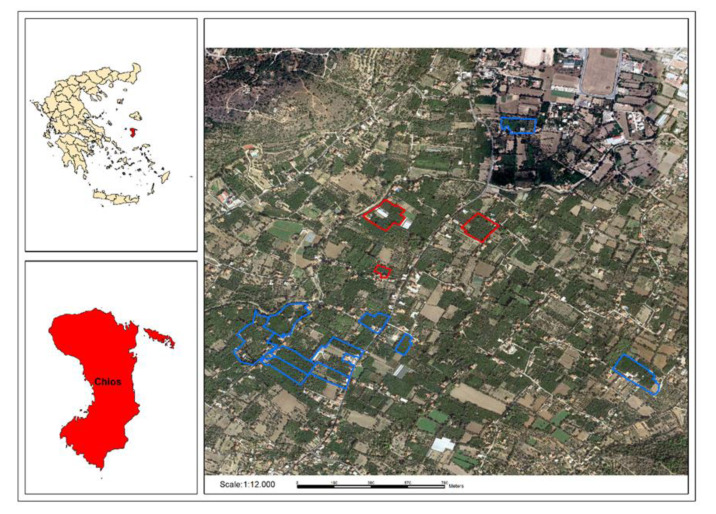
Citrus experimental orchards in Kampos, Chios, Greece. Pilot-scale orchards (1 ha) are marked with red outline and real-scale orchards (10 ha) with blue outline.

Citrus crops play an important role in the sector of the primary production of tree crops in Greece and hold third position after olive and stone trees [34]. Therefore, citrus orchards that are mainly planted with oranges and mandarins were selected in the area of Kampos for the experimental part of the project in a total of 12 ha, as presented in Figure 1.

### 2.2. Selection of Analyzed Pesticides

The scope of the analytical methods applied in the present study comprised almost all ppps authorized for citrus cultivations, besides dithiocarbamates, the herbicide glyphosate and some other active substances, which were analyzed using a single residue method. Throughout the 5 years of the project, the list of compounds reported above was continuously updated, based on the new authorizations granted in the field of citrus crops [13]. Active compounds that were no longer registered were not excluded from the method’s scope, with the objective of examining inappropriate uses as well. Apart from the analysis of the parent compounds of the pesticides, analysis was also conducted on their metabolites, which, according to Regulation 396/2005, are also included in the residue definition for monitoring residues from each pesticide. Finally, a list of 353 active substances and metabolites of pesticides were selected, comprising mainly the chemical classes of amides, carbamates, organophosphates, organochlorines, pyrethroids, sulfonylureas, strobylourines, triazines and dinitroanilines.

### 2.3. Sampling of Plant Products

The objective of the sampling of citrus fruits was to monitor the magnitude and nature of pesticide residues in the citrus experimental orchards in Kampos. Samples of citrus fruits were collected from all the citrus farms involved in each phase of the project. Following the approach reported in the LIFE-BIODELEAR project, the orchards were divided into pilot- (1 ha) and real-scale ones (10 ha) (Figure 1). In both action periods, a similar procedure was followed with respect to sampling, namely samplings for preparatory monitoring (PM) (with the aim of monitoring the existing situation of contamination by pesticide residues prior to the use of the attractant Biodelear) and confirmatory monitoring (CM) (after the implementation of mass trapping with Biodelear).

A combined sample of oranges and mandarins was collected from a subplot of five trees. According to the Commission Directive, 2002/63/EC (2002) [35], citrus fruits belong to the category of medium-sized fresh products (units 25 to 250 g) [35] and, therefore, at least 1 kg was sampled. Every sample was clearly labeled and sent directly to the laboratory within 24 h. Samples were stored in a cool dark place and then dispatched.

### 2.4. Analytical Methodology

#### 2.4.1. Chemicals and Reagents

All pesticide reference standards were of purity above 98% and were purchased from Ehrenstorfer GmbH (Augsburg, Germany), Sigma-Aldrich (St. Louis, Missouri, USA) and Chemservice (West Chester, PA, USA). All the solvents, namely acetonitrile, methanol and water (Fisher Chemical, Loughborough, UK), were of HPLC grade. Primary secondary amine (PSA, 40 lm, Bondesil) sorbent was purchased from Varian Inc. (Palo Alto, CA, USA), while magnesium sulphate (dried) was purchased from Acros Organics (Geel, Belgium).

#### 2.4.2. Standard Solutions

The stock solutions of the individual pesticide standards were prepared at 1000 μg/L in acetone and stored at 20 °C. Composite working solutions at 10 mg/L were also prepared in acetonitrile, separately for GC-amenable pesticides and for LC-amenable pesticides, and stored at 20 °C. All working standard solutions used in the study were derived from the above solutions. Additionally, matrix-matched calibration standards were prepared within the range of 0.01–0.5 μg/mL by serial dilution of orange extract.

#### 2.4.3. Sample Preparation

The detection of pesticide residues in citrus fruits was performed by applying the QuEChERS method [36,37]. According to this method, “ten (10 ± 0.1) g of homogenized samples were weighted in a 50 mL centrifuge tube and extracted using 10 mL of acetonitrile for 1 min, while vigorously shaking by hand. After the addition of the four QuEChERs salts (4 g magnesium sulfate anhydrous, 1 g sodium chloride, 1 g trisodium citrate dihydrate, and 0.5 g disodium hydrogen citrate sesquihydrate), the mixture was shaken intensively and centrifuged for phase separation at 4000 rpm for 5 min. An aliquot (6 mL) of the organic phase was transferred to a 15 mL single-use centrifuge tube containing 150 mg of primary secondary amine and 900 mg of MgSO4, which were used for the cleanup in order to remove interferences and reduce contamination of the instrument, and was shaken vigorously for 1 min. Following an additional centrifugation step, the supernatant was transferred into a screw-cap storage vial”. This final extract was stored in the freezer up until the analysis in the chromatographic systems of gas chromatography with an electron capture detector (GC–ECD) and mass spectrometry (GC–MS), and liquid chromatography triple quadrupole mass spectrometry (LC–MS/MS).

With respect to the detection of residues of plant growth regulators and acidic pesticides, the QuEChERs method combined with alkaline hydrolysis [38,39] was applied for sample preparation. “Five (5 ± 0.1) g of homogenized samples were weighted in a 50 mL centrifuge vial following the addition of 300 µL of a 5 N NaOH solution (this brings pH to a value of ca. 12) and a vigorous 1-min shake of the vial followed. The mixture was allowed to stand for 30 min, with occasional shaking, and 300 µL of a 5N H_2_SO4 solution was added as a neutralization step. The samples were first extracted with 10 mL of acetonitrile for 1 min, while vigorously shaking by hand. Following the addition of the four QuEChERs salts (4 g magnesium sulfate anhydrous, 1 g sodium chloride, 1 g trisodium citrate dihydrate, and 0.5 g disodium hydrogen citrate sesquihydrate), the mixture was shaken intensively and centrifuged for phase separation at 4000 rpm for 5 min. An aliquot (7 mL) of the organic phase was transferred to a 15 mL single-use centrifuge tube containing 150 mg of primary secondary amine and 900 mg of MgSO4, which were used for the cleanup in order to remove interferences and reduce contamination of the instrument, and was shaken vigorously for 1 min. Following an additional centrifugation step, the final extract was transferred into a screw-cap storage vial and stored in the freezer up until the analysis in the chromatographic systems of GC–ECD, GC–MS and LC–MS/MS”.

#### 2.4.4. Determination of Compounds—Instrumentation

##### Analysis of Samples with Gas Chromatography

*GC–ECD analysis* [40]. Two Agilent 6890 chromatographic systems (Agilent Technologies, Santa Clara, CA, USA) connected to an ECD detector functioning in splitless injector mode were employed. Analytes were separated with a HP-5 analytical column (30 m, 0.32 mm i.d. and 0.25 μm film thickness) and confirmed with a DB-17MS column (30 m, 0.32 mm i.d. and 0.25 μm film thickness). Both systems were equipped with the Chemstation chromatography manager and processing software. The helium carrier gas flow rate was 1.5 mL/min for both columns. The temperature of the injectors was set at 230 °C and the splitless injection was carried out with the purge valve closed for 1 min. Injection volume was set at 1 μL. A GC–MS system was also involved in the analysis, with the aim of providing additional verification.

*GC–MS analysis*. A Shimadzu QP 2010 Plus gas chromatography system (Shimadzu, Kyoto, Japan) that was coupled to a mass spectrometer was also used. The GC was equipped with a split/splitless autoinjector (AOC-20i) operating in splitless mode and an autosampler AOC-20s. The analytical capillary column was DB-5MS+DG (30 m × 0.25 mm i.d., 0.25 μm film thickness). Injection volume was set at 1 μL and the injector temperature at 230 °C. The oven temperature was programmed to hold for 1 min at 70 °C, ramp at 45 °C/min to 180 °C, then increased to 230 °C at 1.8 °C /min and then increased to 280 °C at 30 °C /min. The carrier gas was He at 1.4 mL/min. The ion source was set at 200 °C and the interface at 280 °C. The total GC run time was 65 min. Confirmation of the determined analytes was based on the criteria of retention time and the ion abundance of 3 selected ions [41].

##### Analysis of Samples with Liquid Chromatography

*LC–MS/MS analysis* [42]. The liquid chromatographic (LC) system used was an Agilent 1200 Series Quaternary system (Agilent Technologies, CA, USA), and chromatographic separation was achieved using a Zorbax Eclipse XDB C_18_ column (15 cm, 2.1 mm i.d., 3.5 μm). The flow rate was set at 0.27 mL/min and the program applied was gradient with a mobile phase consisting of two solvents. Solvent A was water 5 mmol/L ammonium formate, 0.1% formic acid, 0.02% acetonitrile, while solvent B was methanol 5 mmol/L ammonium formate, 0.1% formic acid. In an effort to avoid carryover, the autosampler was purged with a mixture of methanol/water (50/50, *v/v*) before each sample was injected with a 5 mL injection volume. The mass spectrometer used was the Agilent 6410 Triple Quad LC/MS equipped with an electrospray ionization (EI) interface that operated in the positive mode. Capillary voltage (CV) and collision cell energy varied depending on the precursor ion of each analyte: source temperature was set at 300 °C, drying gas flow rate at 11 L/min and nebulizing gas pressure at 40 psi. Instrument control, data acquisition and processing were achieved by means of the Agilent MassHunter software version B.01.04 (B84), which was installed in the instrument. Confirmation of the determined analytes was based on the criteria of retention time and ion abundance of qualitative and quantitative ions, in accordance with European guidelines. The ion transitions used for quantification and qualification purposes, as well as the capillary voltage (CV) and collision cell energy, have been published in previous publications [39,42,43].

### 2.5. Statistical Analysis

The results obtained in the study were analyzed using one-way analysis of variance (one-way ANOVA). All data were analyzed using the statistical package JMP [44].

## 3. Results and Discussion

### 3.1. Method Performance

The analytical methods used have previously been fully validated [34] and accredited for citrus fruits [45]. Therefore, procedural recoveries were performed with each sample batch in order to ensure the performance of the method. In total, two levels of quality control samples (QC) at 0.01 mg/kg (*n* = 3) and 0.1 mg/kg (*n* = 3) were prepared in blank orange samples and analyzed to ensure the validity of the results. Accuracy was expressed as the recovery value as a percentage and the obtained values were found to be in the range of 60–140%, [41]. The limit of quantification (LOQ) was set at 0.01 mg/kg.

### 3.2. Detection of Pesticides

During the implementation of the five-year project, a total of six samplings of citrus fruits were conducted. In total, 511 citrus fruit samples were collected from the pilot-scale experimental orchards and 741 samples from the real-scale experimental orchards. All samples were analyzed using the above-reported analytical methodology to investigate their potential contamination with the target compounds. 

#### 3.2.1. Pesticide Occurrence in Pilot-Scale Experimental Orchards

Preparatory monitoring: Two hundred samples of citrus fruits were collected and analyzed as reported above. No positive detections of pgrs were noted. As regards residues of ppps, the insecticides chlorpyrifos and deltamethrin and the fungicide propamocarb were detected (Table 1).

**Figure 2:**
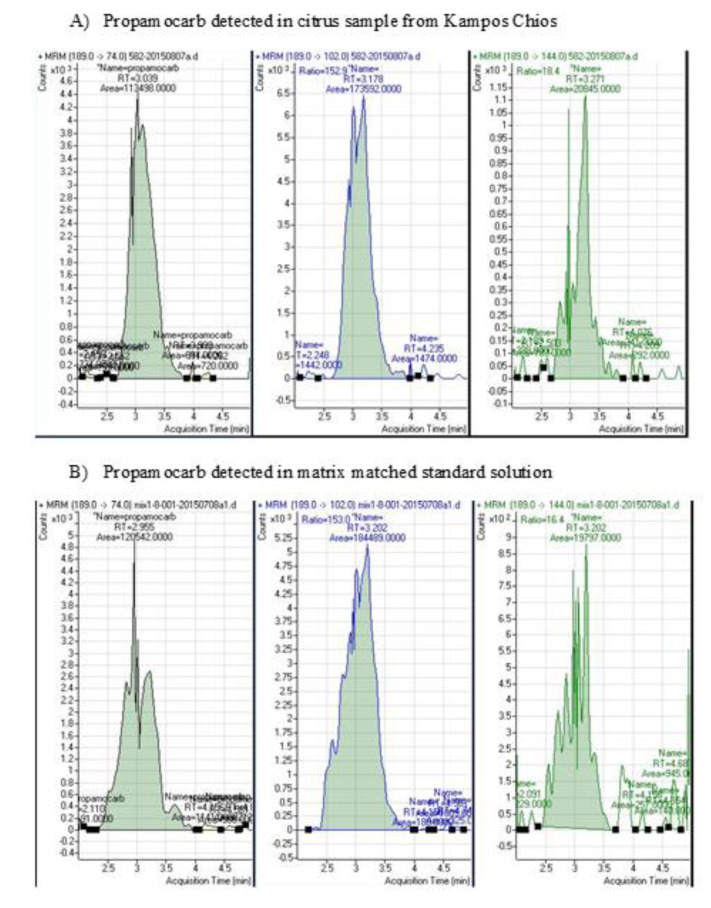
Determination of the fungicide propamocarb in the LC–MS/MS. (**A**) Chromatogram of fragment ions (parent and daughter) of propamocarb detected in citrus sample from Kampos, Chios. (**B**) Chromatogram of fragment ions (parent and daughter) of propamocarb detected in matrix-matched standard solution.

Chlorpyrifos was detected in 58 fruit samples, with the highest calculated concentration reaching 0.35 mg/kg, while deltamethrin was detected in 22 fruit samples, with the highest calculated concentration being that of 0.12 mg/kg. Additionally, eleven citrus fruit samples gave positive detections for propamocarb (Figure 2). The results of the above samplings provided primary information on the status of pesticide residues in the pilot experimental orchards of the studied area.

**Figure 3:**
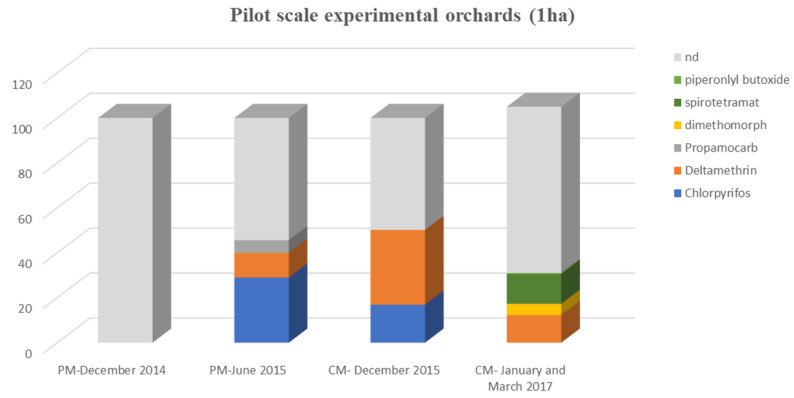
Incidence (% of total samples analyzed) of pesticide residues in citrus fruits sampled from the pilot experimental area in preparatory monitoring (PM) and confirmatory monitoring (CM) stages.

Confirmatory sampling: Three hundred and eleven samples from the same experimental orchards were collected and analyzed as reported above (Figure 3). 

As was the case in the preparatory monitoring phase, no detectable residues of pgrs were determined. A percentage of 26.2% of the analyzed samples gave positive results for deltamethrin (0.002–0.026 mg/kg), 16.9% for chlorpyrifos (0.0021–0.098 mg/kg), 13.2% for spirotetramat (0.0074–0.083 mg/kg) and 5% for dimethomorph (0.0058–0.1 mg/kg), while only one sample gave a positive determination of piperonyl butoxide (Table 1). Residues of the fungicide propamocarb, which was detected during the preparatory monitoring, were not found. 

Deltamethrin is an insecticide and a member of the class of synthetic pyrethroids. It is a non-systemic insecticide with contact and stomach action against a wide range of pests [46]. This pesticide is approved for use in citrus cultivation for the control of medfly [13] and, as observed, it was detected in both PM (11%) and CM (26.2%) samplings, confirming its widespread use. Dimethomorph is a systemic fungicide with good protectant and antisporulant activity, while spirotetramat is an insecticide, applied for the control of aphids, mites and white flies, by inhibiting acetyl-CoA carboxylase [46]. Both compounds have received authorization for application in citrus crops [13]. Their positive detections at 5% and 13.2% in CM could be attributed to increased infestations of fungal or insect pests during the current cultivation period.

Piperonyl butoxide (PBO) is an analyte used as an ingredient in the formulation of ppps. In particular, although it is not effective as a pesticide, it increases the potency of certain pesticides, such as carbamates, pyrethrins, pyrethroids and androtenone [47,48]. Therefore, determination of its residues in a plant commodity provided an indication of potential applications of insecticide in cultivations of this kind.

#### 3.2.2. Pesticide Occurrence in Real-Scale Experimental Orchards

Preparatory monitoring: Out of a total of 341 samples of citrus fruits that were collected, 41% of them showed residues of ppps, whereas 59% of the collected samples showed no detectable residues of ppps (Table 2). No detectable residues of pgrs were determined in any of the samples. 

**Figure 4:**
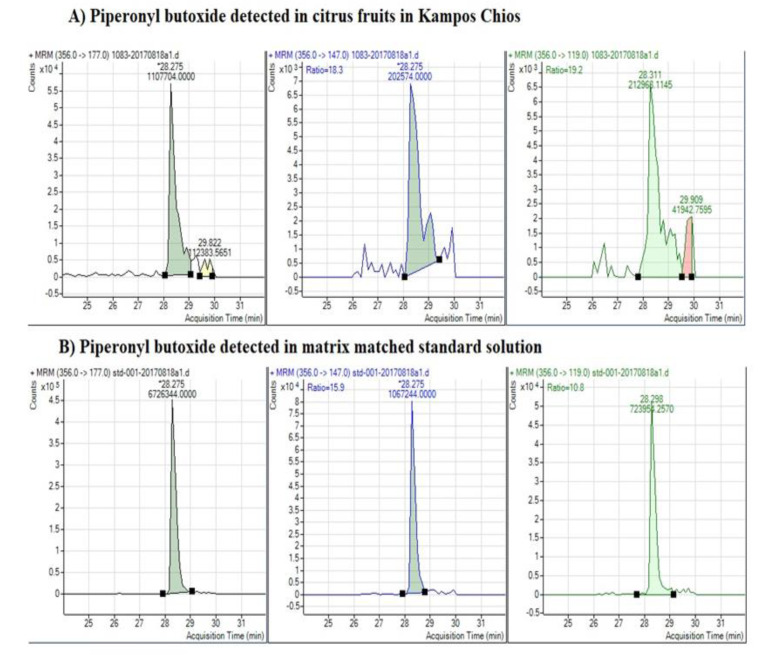
Determination of the synergist piperonyl butoxide in the LC–MS/MS. (**A**) Chromatogram of fragment ions (parent and daughter) of piperonyl butoxide detected in citrus samples from Kampos, Chios. (**B**) Chromatogram of fragment ions (parent and daughter) of piperonyl butoxide detected in matrix-matched standard solution.

According to the obtained results, spirotetramat was detected in 12.9% (0.0048 mg/kg to 0.049 mg/kg), dimethomorph in 3.2% (<0.01 mg/kg), flumioxazine in 1.8% (0.086–0.12 mg/kg), mepanypirim only in one sample (0.0075 mg/kg), azoxystrobin in 1.2% (0.0021–0.0043 mg/kg), propamocarb in 0.9% (0.013–0.033 mg/kg) and the synergist piperonyl butoxide (Figure 4) in 22.3% (0.0033–0.80 mg/kg) (Table 2).

Flumioxazine is a herbicide absorbed by leaves and germinating seedlings, applied for the protection of crops from weeds (annual broad-leaved ones and some annual grasses pre- and post-emergence) [46]. As it has been authorized for application in citrus orchards, the contamination of 1.8% of the citrus samples with this compound may be attributed to herbicidal agricultural practices.

**Figure 5:**
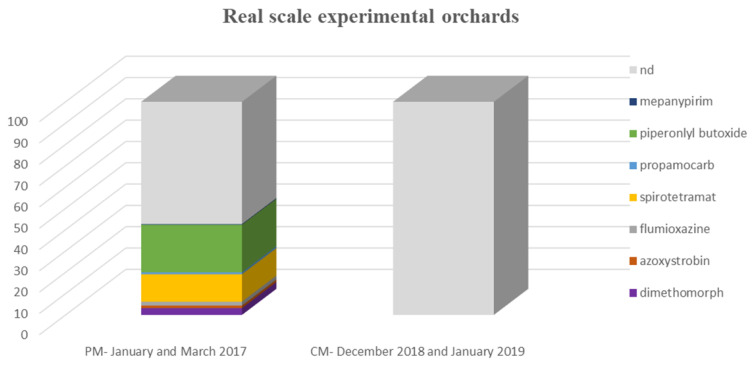
Incidence (% of total samples analyzed) of pesticide residues in citrus fruits sampled from the real-scale experimental area in preparatory monitoring (PM) and confirmatory monitoring (CM) stages.

Confirmatory sampling: A total of 400 samples were collected and analyzed. The completion of sample preparation and analysis showed no detectable residues of ppps or pgrs (Figure 5).

Similar results have also been reported by others. For example, chlorpyrifos and dimethomorph residues were also prevalent in analyzed citrus samples from local markets in Switzerland [19], chlorpyrifos in the Aegean and Mediterranean regions of Turkey [18] and chlorpyrifos and azoxystrobin in China [21].

#### 3.2.3. Citrus Samples with Multiple Residues

Among the citrus samples analyzed in the pilot-scale experiment, there were 17 samples in which two compounds were detected, and six samples in which three compounds were detected (chlorpyrifos, deltamethrin and propamocarb) for the preparatory samplings, while the respective values were 31 (2 compounds) and 4 (3 compounds) in the confirmatory samplings. With respect to the real-scale experiment, 12 samples gave positive determinations of two compounds (spirotetramat was identified in all of them) and one sample gave positive determinations of three compounds (spirotetramat, dimethomorph and piperonyl butoxide).

Among the unprocessed plant products analyzed in the framework of the European Union Control Program (EUCP) during the five-year duration of the study [49,50,51,52,53], multiple residues were also reported in citrus samples: in 2014, up to 60.5% (nine different pesticides were detected in an individual orange sample); in 2015, up to 5.6% (seven different compounds were determined in an individual sample of orange juice) and in 2017, up to 58.7% (in two orange samples originating from a third country, up to 12 different active compounds were reported, while in two other samples, which were produced within the EU, 11 pesticide residues were determined in each of them). In all reports, chlorpyrifos was among the most frequently quantified pesticides.

#### 3.2.4. Citrus Samples with Residues of Non-Authorized Pesticides

Among other substances, positive residue detections were also obtained for non-authorized pesticides (Table 3). 

Chlorpyrifos is no longer approved at EU level [54] and is, therefore, not authorized for use in all crops. Citrus fruits were found to be contaminated with chlorpyrifos only in the pilot scale, in 29% of the samples in PM and in 16.9% in the CM. After the implementation of the developed IPMS for the control of medfly in the real-scale experimental orchards, it was not detected.

Chlorpyrifos residues were also predominant in grape samples originating from local markets in Turkey [61], Croatia and Slovenia [62] and Algeria [63], as well as in immature citrus fruits (putgyul) collected directly from orchards in Korea [64], in a study that followed an experimental approach similar to the present study as regards samplings before harvest.

Propamocarb, azoxystrobin and mepanypirim are not permitted for use in citrus cultivation in Greece [13]. All three compounds were detected in the preparatory monitoring stages of the project and not in the corresponding confirmatory stage. Strobilurin fungicide azoxystrobin shows systemic translaminar and protectant action and possesses additional curative and eradicant properties [46]. It was only detected in the PM of the real-scale experiment in 1.2% of the samples analyzed. Similarly, mepanypirim is a non-systemic fungicide with preventive action, detected at 0.3% only in the PM of the real-scale experiment. Their positive detection could possibly be attributed either to the potential drifting of spaying solution through neighboring orchards (environmental contaminant) or to inappropriate use in the fields of the study. Especially with regard to the fungicide propamocarb, it was detected in 5.55% of the samples in PM and 0.9% in CM. Its detection (Figure 2) may also be attributed to its actual application on vegetables that were cultivated under the canopy of citrus trees (co-cultivation) in the specific citrus orchards. This assumption is further confirmed by its repeated detection in PM and CM.

The concentrations determined cannot be compared with the MRLs set by legislation (Table 3) for each compound, since, according to Regulation 396/2005, these legal limits are set for the final product that will be distributed to the market and offered for consumption. The citrus fruits of the present study were sampled from citrus trees at various growth (BBCH) stages. However, and especially with respect to chlorpyrifos, which is not authorized in the EU, based on EFSA Annual Reports on pesticide residues in the last five years [49,50,51,52,53], several MRL exceedances for chlorpyrifos in oranges analyzed in the EU have been reported.

### 3.3. Seasonal Distribution and Variation

Maximum concentration levels were calculated in summer samplings, which was obviously to be expected, since the majority of applications for the control of *C. capitata* in citrus crops take place in late spring and early summer. Lower concentration levels were observed in winter samplings, mainly due to chemical degradation (photolysis, hydrolysis or oxidation) and the biodegradation of the compounds since they were applied in the field [65].

The results obtained from the summer and winter samplings for the two insecticides that were found in both cases (chlorpyriphos and deltamethrin) within the pilot-scale experiments were compared with one-way ANOVA. The results showed a significant difference between summer (PM) and winter (CM) samplings (chlorpyrifos: F = 59.3; df = 1; *p* < 0.05 and deltamethrin F = 9.7; df = 1; *p* < 0.05). Since no residues were found in the winter samplings at the real scale, a statistical analysis of the results was not required.

### 3.4. Annual Distribution and Variation

Mean annual concentrations for all combinations of pesticide/samplings were calculated for all five years. Results (Table 1 and Table 2) showed a reduction in absolute numbers per compound throughout the 2014 to 2018 period, leading to the conclusion that the developed strategy based on the attractant Biodelear had a positive effect on the chemical contamination of citrus fruits. It has thus become apparent that the five-year period of the project implementation allowed sufficient time for the improvement of the environmental quality standard of citrus orchards in which intensive agricultural practices had been carried out in the past.

## 4. Conclusions

This 5-year monitoring survey indicated that the analysis of 1252 citrus fruit samples collected from 12 orchards gave positive determinations of one or multiple pesticide residues, mainly in preparatory monitoring samples. Our findings mainly comprised insecticides, fungicides and a synergist, while three of the detected pesticides have either been banned or are not authorized for use in citrus fruits. The most frequently detected compounds were chlorpyrifos, deltamethrin, spirotetramat and pyperonyl butoxide. The analysis of pesticide residues of sampled citrus fruits in the present study provided collateral confirmation to the LIFE-BIODELEAR project so as to evaluate the effectiveness of the attractant in the control of medfly. Furthermore, on completion of the project and during the real-scale confirmatory samplings, the absence of pesticide residues indicates, among other parameters, the improvement of the environmental quality of the studied area and of food safety. The study clearly demonstrates that alternative, environmentally friendly approaches, such as those developed and used in our study, reduce the use of pesticides and therefore contribute to a healthier agroecosystem, with safer agricultural products for consumers.

## Figures and Tables

**Table 1:** Occurrence of pesticide residues in citrus fruits of Kampos, Chios in Greece in pilot area citrus orchards.

Sampling/Experimental Area	Pesticide	No of Positive Samples (%)	Concentration Range (mg/kg)	Mean Value (mg/kg)	No. (%) of Exceedance
PM ^a^ ^(December 2014, June 2015)^	Chlorpyrifos	58 (29%)	0.0041–0.35	0.073	86.2%
	Deltamethrin	22 (11%)	0.0052–0.12	0.0702	36.4%
	Propamocarb	11 (5.55%)	0.0047–0.007	0.073	-
CM ^b^ ^(December 2015, January–March 2017)^	Dimethomorph	11 (5%)	0.0058–0.1004	0.029	-
	Chlorpyrifos	37 (16.9%)	0.0021–0.098	0.025	81.1%
	Deltamethrin	57 (26.2%)	0.002–0.026	0.024	-
	Spirotetramat	29 (13.2%)	0.0074–0.083	0.03	-
	Piperonyl butoxide	1 (0.45%)	0.0049	-	-

^a^ PM: Preparatory monitoring, ^b^ CM: Confirmatory monitoring.

**Table 2:** Occurrence of pesticide residues in citrus fruits of Kampos, Chios in Greece in real-scale area citrus orchards.

Sampling/Experimental Area	Pesticide	No of Positive Samples (%)	Concentration Range (mg/kg)	Mean Value (mg/kg)	No. (%) of Exceedance
PM ^a^ ^(January–March 2017)^	Dimethomorph	11 (3.2%)	0.0026–0.0061	0.032	-
	Azoxystrobin	4 (1.2%)	0.0021–0.0043	0.0033	-
	Flumioxazine	6 (1.8%)	0.086–0.12	0.1	100%
	Spirotetramat	44 (12.9%)	0.0048–0.049	0.05	-
	Propamocarb	3 (0.9%)	0.0132–0.033	0.026	100%
	Piperonyl butoxide	76 (22.3%)	0.0033–0.8013	0.048	-
	Mepanypirim	1 (0.3%)	0.0075	-	-
CM ^b^ ^(December 2018–January 2019)^	nd	-	-	-	-

^a^ PM: Preparatory monitoring, ^b^ CM: Confirmatory monitoring.

**Table 3:** Type, maximum residue limits (MRLs) and authorization in citrus crops of the pesticides detected in the experimental citrus orchards.

Pesticide	Type	MRL (mg/kg)	Legislation of MRLs	Authorization in Citrus Crops
Chlorpyrifos	Insecticide	0.01	Reg. (EU) 2020/1085 [54]	No
Deltamethrin	Insecticide	0.04	Reg. (EU) 2018/832 [55]	Yes
Propamocarb	Fungicide	0.01	Reg. (EU) 2020/856 [12]	No
Dimethomorph	Fungicide	0.8	Reg. (EU) 2016/1902 [56]	Yes
Spirotetramat	Insecticide	1	Reg. (EU) 2019/1015 [57]	Yes
Piperonyl butoxide	Synergist	-	-	-
Azoxystrobin	Fungicide	15	Reg. (EU) 2019/552 [58]	No
Flumioxazine	Herbicide	0.02	Reg. (EU) No 2014/1126 [59]	Yes
Mepanypirim	Fungicide	0.01	Reg. (EU) 2016/486 [60]	No

## Data Availability

Not applicable.
